# The genome sequence of the Green Silver-lines, *Pseudoips prasinana *(Linnaeus, 1758)

**DOI:** 10.12688/wellcomeopenres.21014.1

**Published:** 2024-03-01

**Authors:** Douglas Boyes, Peter W.H. Holland, University of Oxford and Wytham Woods Genome Acquisition Lab, Darwin Tree of Life Barcoding collective, Wellcome Sanger Institute Tree of Life Management, Samples and Laboratory team, Wellcome Sanger Institute Scientific Operations: Sequencing Operations, Wellcome Sanger Institute Tree of Life Core Informatics team, Tree of Life Core Informatics collective, Darwin Tree of Life Consortium

**Affiliations:** 1 UK Centre for Ecology & Hydrology, Wallingford, England, UK; 2 University of Oxford, Oxford, England, UK

**Keywords:** Pseudoips prasinana, Green Silver-lines, genome sequence, chromosomal, Lepidoptera

## Abstract

We present a genome assembly from an individual female *Pseudoips prasinana* (the Green Silver-lines; Arthropoda; Insecta; Lepidoptera; Nolidae). The genome sequence is 1,125.7 megabases in span. Most of the assembly is scaffolded into 33 chromosomal pseudomolecules, including the Z and W sex chromosomes. The mitochondrial genome has also been assembled and is 15.23 kilobases in length. Gene annotation of this assembly on Ensembl identified 20,065 protein coding genes.

## Species taxonomy

Eukaryota; Opisthokonta; Metazoa; Eumetazoa; Bilateria; Protostomia; Ecdysozoa; Panarthropoda; Arthropoda; Mandibulata; Pancrustacea; Hexapoda; Insecta; Dicondylia; Pterygota; Neoptera; Endopterygota; Amphiesmenoptera; Lepidoptera; Glossata; Neolepidoptera; Heteroneura; Ditrysia; Obtectomera; Noctuoidea; Nolidae; Chloephorinae; *Pseudoips*; *Pseudoips prasinana* (Linnaeus, 1758) (NCBI:txid2758353).

## Background

The Green Silver-lines *Pseudoips prasinana* is a nocturnal moth in the family Nolidae, superfamily Noctuioidea, found in woodland habitats across the Palaearctic from Portugal to Japan ( GBIF Secretariat, 2023). The adult has orange antennae, pink front legs and bright green forewings crossed by a series of silver diagonal lines. Adults of *P. prasinana* are attracted to light, and can also be found resting in the foliage of trees or on low growing woodland plants ( South, 1961). In Britain, the species is widely distributed across England and Wales, but more local in Scotland and Northern Ireland ( NBN Atlas Partnership, 2023). It is locally common in Ireland ( MothsIreland, 2024).

In Britain, adults of *P. prasinana* have been recorded primarily from May to June ( NBN Atlas Partnership, 2023), with larvae developing through late summer and early autumn. The larvae feed on foliage of oak *Quercus* spp. and beech *Fagus sylvatica* trees, with pupation occurring inside a silken cocoon spun by the larva on the underside of leaves. The pupal stage overwinters. The similarity of the cocoon to those produced by silkmoths has prompted research into biochemistry of *P. prasinana* cocoon silks. A combination of X-ray analysis, proteomics and larval transcriptomics has revealed that *P. prasinana* produces some silk proteins with similar core amino acid composition to those of *Bombyx mori*, plus many additional silk proteins ( Rindos *et al.*, 2021).

Male *P. prasinana* produce ultrasonic and audible clicks from tymbal organs in a cleft on the second abdominal segment; these consist of a disk of flexible cuticle, an air-filled cavity and large fan-shaped muscles ( Skals & Surlykke, 1999). The sound-producing organ is only found in males suggesting a role in sexual communication rather than as a bat defence system.

A genome sequence of the Green Silver-lines *Pseudoips prasinana* was determined as part of the Darwin Tree of Life project. The genome sequence will facilitate research into host plant specialisation and silk biochemistry, and will contribute to the growing set of resources for studying molecular evolution in insects.

## Genome sequence report

**Figure 1.**
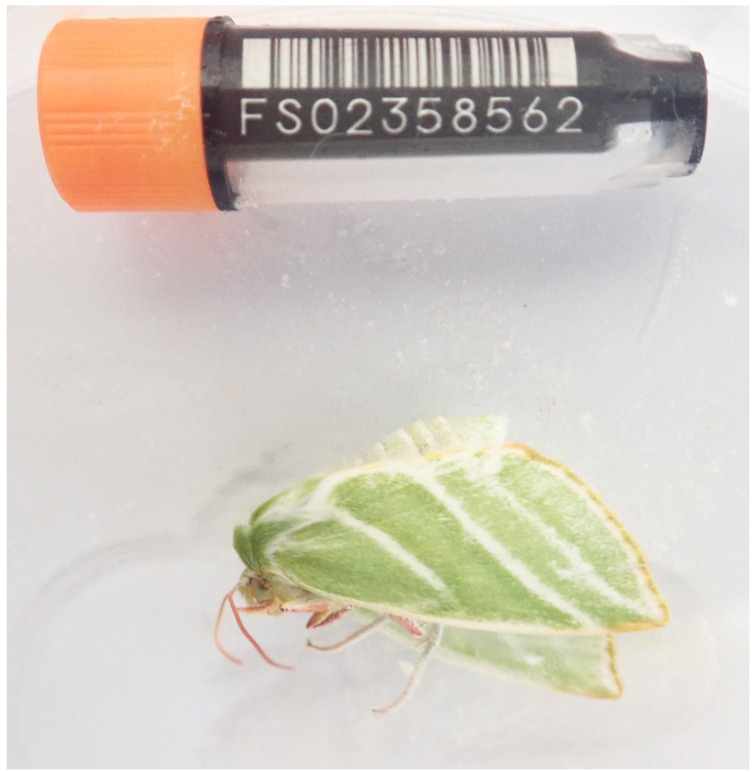
Photograph of the *Pseudoips prasinana* (ilPsePras1) specimen used for genome sequencing.

The genome was sequenced from one female *Pseudoips prasinana* ( Figure 1) collected from Wytham Woods, Oxfordshire, UK (51.77, –1.34). A total of 33-fold coverage in Pacific Biosciences single-molecule HiFi long reads was generated. Primary assembly contigs were scaffolded with chromosome conformation Hi-C data. Manual assembly curation corrected 41 missing joins or mis-joins and removed 14 haplotypic duplications, reducing the assembly length by 0.73% and the scaffold number by 26.32%, and decreasing the scaffold N50 by 1.03%.

**Figure 2.**
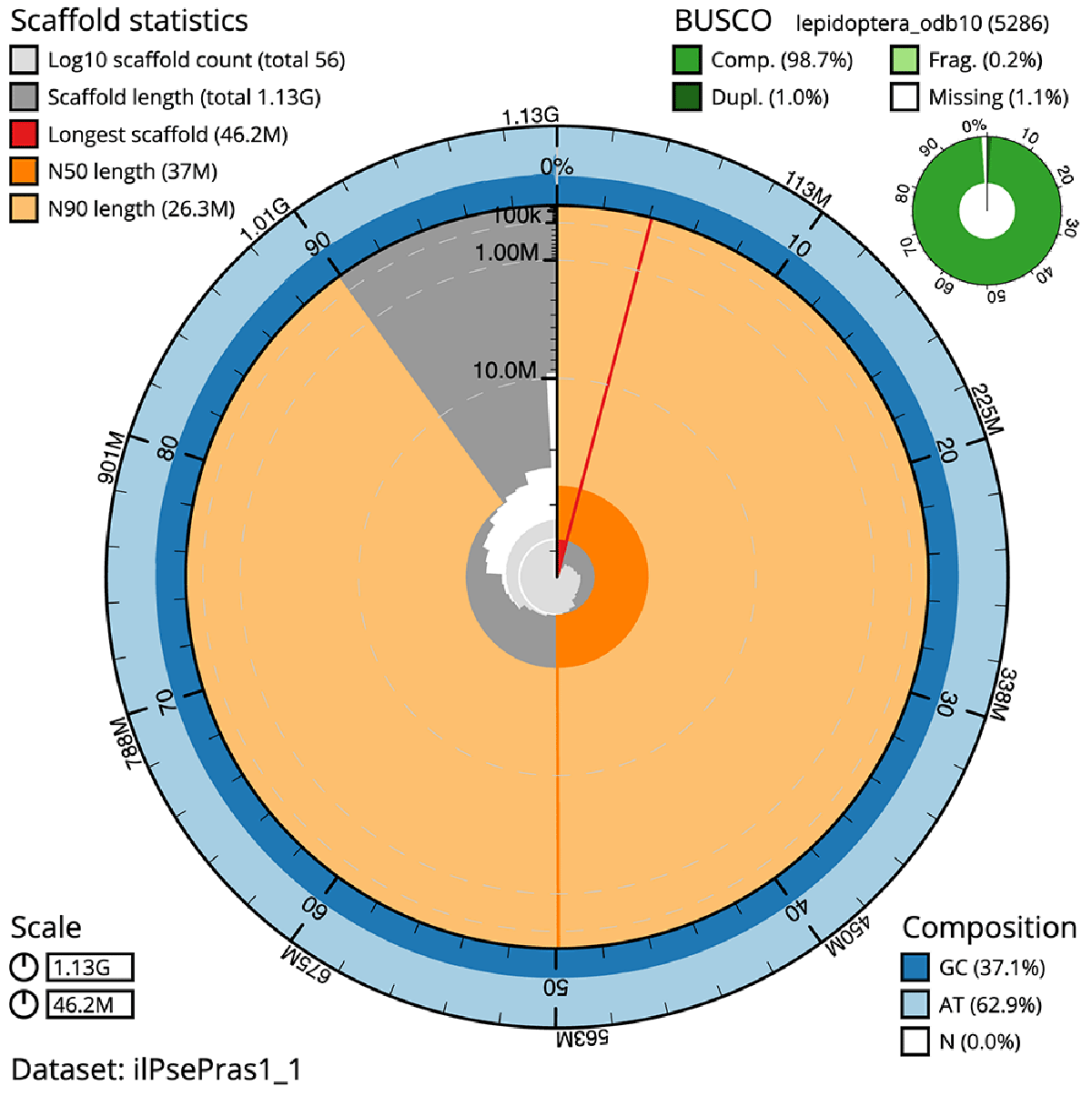
Genome assembly of *Pseudoips prasinana*, ilPsePras1.1: metrics. The BlobToolKit Snailplot shows N50 metrics and BUSCO gene completeness. The main plot is divided into 1,000 size-ordered bins around the circumference with each bin representing 0.1% of the 1,125,692,588 bp assembly. The distribution of scaffold lengths is shown in dark grey with the plot radius scaled to the longest scaffold present in the assembly (46,196,481 bp, shown in red). Orange and pale-orange arcs show the N50 and N90 scaffold lengths (36,985,250 and 26,292,167 bp), respectively. The pale grey spiral shows the cumulative scaffold count on a log scale with white scale lines showing successive orders of magnitude. The blue and pale-blue area around the outside of the plot shows the distribution of GC, AT and N percentages in the same bins as the inner plot. A summary of complete, fragmented, duplicated and missing BUSCO genes in the lepidoptera_odb10 set is shown in the top right. An interactive version of this figure is available at https://blobtoolkit.genomehubs.org/view/ilPsePras1_1/dataset/ilPsePras1_1/snail.

**Figure 3.**
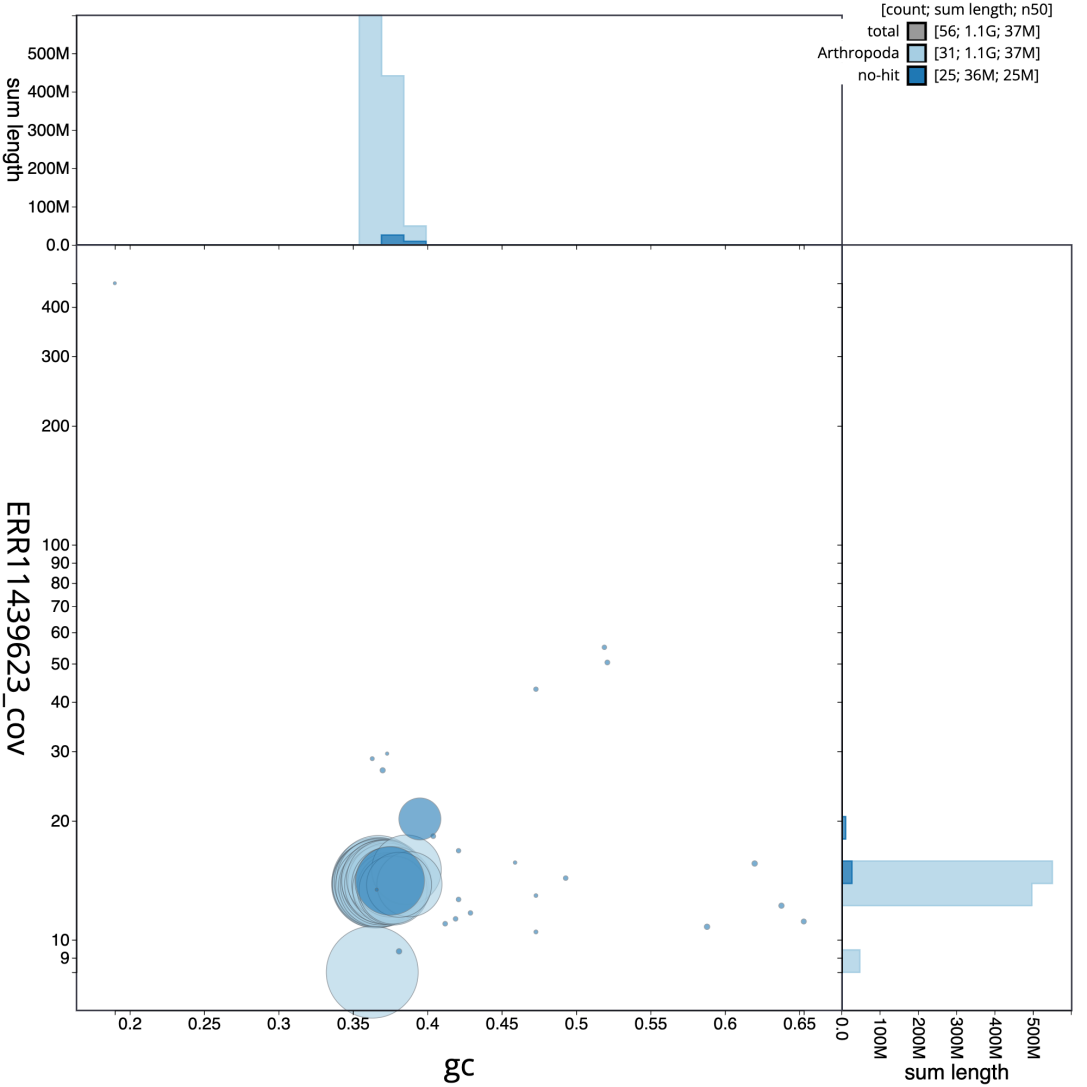
Genome assembly of *Pseudoips prasinana*, ilPsePras1.1: BlobToolKit GC-coverage plot. Scaffolds are coloured by phylum. Circles are sized in proportion to scaffold length. Histograms show the distribution of scaffold length sum along each axis. An interactive version of this figure is available at https://blobtoolkit.genomehubs.org/view/ilPsePras1_1/dataset/ilPsePras1_1/blob.

**Figure 4.**
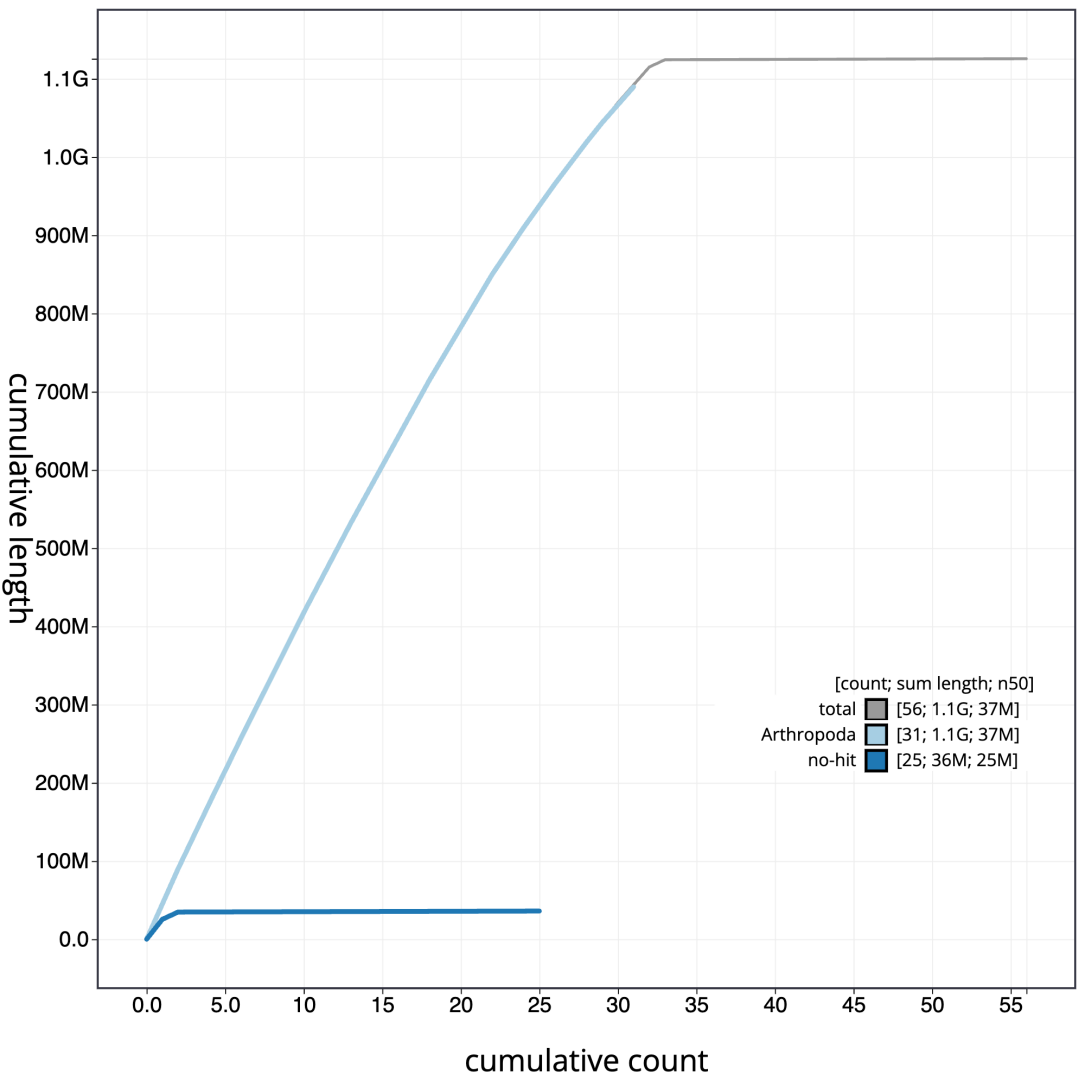
Genome assembly of *Pseudoips prasinana*, ilPsePras1.1: BlobToolKit cumulative sequence plot. The grey line shows cumulative length for all scaffolds. Coloured lines show cumulative lengths of scaffolds assigned to each phylum using the buscogenes taxrule. An interactive version of this figure is available at https://blobtoolkit.genomehubs.org/view/ilPsePras1_1/dataset/ilPsePras1_1/cumulative.

**Figure 5.**
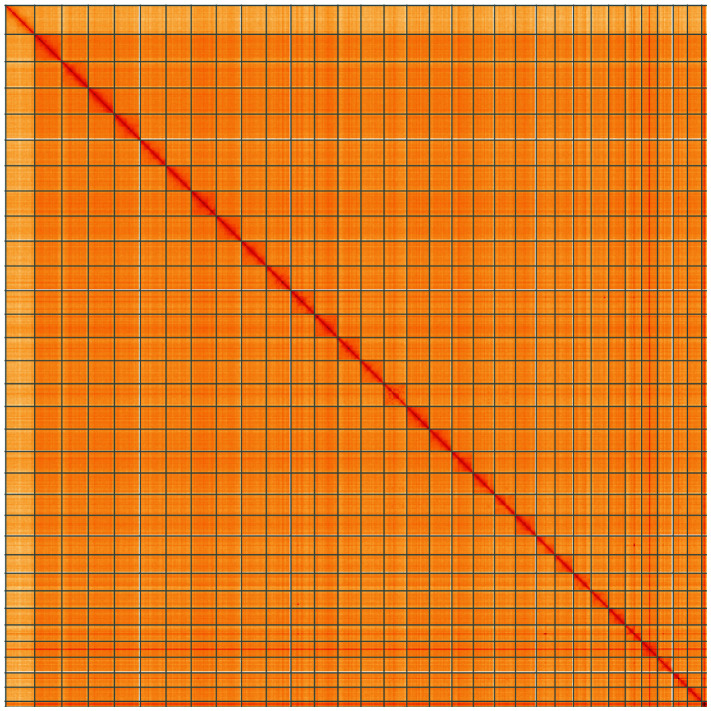
Genome assembly of *Pseudoips prasinana*, ilPsePras1.1: Hi-C contact map of the ilPsePras1.1 assembly, visualised using HiGlass. Chromosomes are shown in order of size from left to right and top to bottom. An interactive version of this figure may be viewed at https://genome-note-higlass.tol.sanger.ac.uk/l/?d=SgMxdIG5RIWvn6p8zxCpMQ.

The final assembly has a total length of 1125.7 Mb in 55 sequence scaffolds with a scaffold N50 of 37.0 Mb ( Table 1). The snailplot in Figure 2 provides a summary of the assembly statistics, while the distribution of assembly scaffolds on GC proportion and coverage is shown in Figure 3. The cumulative assembly plot in Figure 4 shows curves for subsets of scaffolds assigned to different phyla. Most (99.88%) of the assembly sequence was assigned to 33 chromosomal-level scaffolds, representing 31 autosomes and the Z and W sex chromosomes. Chromosome-scale scaffolds confirmed by the Hi-C data are named in order of size ( Figure 5; Table 2). While not fully phased, the assembly deposited is of one haplotype. Contigs corresponding to the second haplotype have also been deposited. The mitochondrial genome was also assembled and can be found as a contig within the multifasta file of the genome submission.

**Table 1.** Genome data for *Pseudoips prasinana*, ilPsePras1.1.

Project accession data		
Assembly identifier	ilPsePras1.1	
Species	*Pseudoips prasinana*	
Specimen	ilPsePras1	
NCBI taxonomy ID	2758353	
BioProject	PRJEB61694	
BioSample ID	SAMEA7631558	
Isolate information	ilPsePras1, female: abdomen (DNA sequencing), thorax (DNA and Hi-C sequencing)	
**Assembly metrics** *		*Benchmark*
Consensus quality (QV)	66.0	*≥ 50*
*k*-mer completeness	100.0%	*≥ 95%*
BUSCO **	C:98.7%[S:97.7%,D:1.0%], F:0.2%,M:1.1%,n:5,286	*C ≥ 95%*
Percentage of assembly mapped to chromosomes	99.88%	*≥ 95%*
Sex chromosomes	ZW	*localised homologous pairs*
Organelles	Mitochondrial genome: 15.23 kb	*complete single alleles*
Raw data accessions		
PacificBiosciences SEQUEL II	ERR11279101, ERR11279102	
Hi-C Illumina	ERR11439624	
Genome assembly		
Assembly accession	GCA_951640165.1	
*Accession of alternate haplotype*	GCA_951640185.1	
Span (Mb)	1,125.7	
Number of contigs	105	
Contig N50 length (Mb)	25.1	
Number of scaffolds	55	
Scaffold N50 length (Mb)	37.0	
Longest scaffold (Mb)	46.2	
Genome annotation		
Number of protein-coding genes	20,065	
Number of gene transcripts	20,235	

* Assembly metric benchmarks are adapted from column VGP-2020 of “Table 1: Proposed standards and metrics for defining genome assembly quality” from ( Rhie *et al.*, 2021). ** BUSCO scores based on the lepidoptera_odb10 BUSCO set using version 5.3.2. C = complete [S = single copy, D = duplicated], F = fragmented, M = missing, n = number of orthologues in comparison. A full set of BUSCO scores is available at https://blobtoolkit.genomehubs.org/view/ilPsePras1_1/dataset/ilPsePras1_1/busco.

**Table 2.** Chromosomal pseudomolecules in the genome assembly of *Pseudoips prasinana*, ilPsePras1.

INSDC accession	Chromosome	Length (Mb)	GC%
OX621235.1	1	43.7	36.5
OX621236.1	2	42.3	36.5
OX621237.1	3	41.87	36.5
OX621238.1	4	41.59	36.5
OX621239.1	5	41.12	36.5
OX621240.1	6	40.31	37.0
OX621241.1	7	40.25	37.0
OX621242.1	8	40.19	36.5
OX621243.1	9	39.93	36.5
OX621244.1	10	39.44	36.5
OX621245.1	11	37.87	36.5
OX621246.1	12	37.38	37.0
OX621247.1	13	36.99	36.5
OX621248.1	14	36.95	37.0
OX621249.1	15	36.43	37.0
OX621250.1	16	36.36	37.0
OX621251.1	17	35.89	37.0
OX621252.1	18	34.47	37.0
OX621253.1	19	34.06	37.0
OX621254.1	20	33.58	37.5
OX621255.1	21	33.24	37.0
OX621256.1	22	30.02	38.0
OX621257.1	23	29.59	37.5
OX621258.1	24	28.23	37.5
OX621259.1	25	28.02	37.5
OX621260.1	26	26.67	37.5
OX621261.1	27	26.29	38.5
OX621262.1	28	25.46	37.5
OX621263.1	29	25.06	37.5
OX621264.1	30	22.89	39.0
OX621265.1	31	22.78	38.0
OX621266.1	W	9.3	39.5
OX621234.1	Z	46.2	36.5
OX621267.1	MT	0.02	19.5

The estimated Quality Value (QV) of the final assembly is 66.0 with *k*-mer completeness of 100.0%, and the assembly has a BUSCO v5.3.2 completeness of 98.7% (single = 97.7%, duplicated = 1.0%), using the lepidoptera_odb10 reference set ( *n* = 5,286).

Metadata for specimens, barcode results, spectra estimates, sequencing runs, contaminants and pre-curation assembly statistics are given at https://links.tol.sanger.ac.uk/species/2758353.

## Genome annotation report

The *Pseudoips prasinana* genome assembly (GCA_951640165.1) was annotated at the European Bioinformatics Institute (EBI) using the Ensembl rapid annotation pipeline ( Table 1; https://rapid.ensembl.org/Pseudoips_prasinana_GCA_951640165.1/Info/Index). The resulting annotation includes 20,235 transcribed mRNAs from 20,065 protein-coding genes.

## Methods

### Sample acquisition and nucleic acid extraction

A female *Pseudoips prasinana* (specimen ID Ox000409, ToLID ilPsePras1) was collected from Wytham Woods, Oxfordshire (biological vice-county Berkshire), UK (latitude 51.77, longitude –1.34) on 2020-05-22 using a light trap. The specimen was collected and identified by Douglas Boyes (University of Oxford) and preserved on dry ice.

The workflow for high molecular weight (HMW) DNA extraction at the Wellcome Sanger Institute (WSI) includes a sequence of core procedures: sample preparation; sample homogenisation, DNA extraction, fragmentation, and clean-up. In sample preparation, the ilPsePras1 sample was weighed and dissected on dry ice ( Jay *et al.*, 2023). Tissue from the thorax and abdomen was homogenised using a PowerMasher II tissue disruptor ( Denton *et al.*, 2023a). HMW DNA was extracted using the Automated MagAttract v1 protocol ( Sheerin *et al.*, 2023). DNA was sheared into an average fragment size of 12–20 kb in a Megaruptor 3 system with speed setting 30 ( Todorovic *et al.*, 2023). Sheared DNA was purified by solid-phase reversible immobilisation ( Strickland *et al.*, 2023): in brief, the method employs a 1.8X ratio of AMPure PB beads to sample to eliminate shorter fragments and concentrate the DNA. The concentration of the sheared and purified DNA was assessed using a Nanodrop spectrophotometer and Qubit Fluorometer and Qubit dsDNA High Sensitivity Assay kit. Fragment size distribution was evaluated by running the sample on the FemtoPulse system.

Protocols developed by the WSI Tree of Life laboratory are publicly available on protocols.io ( Denton *et al.*, 2023b).

### Sequencing

Pacific Biosciences HiFi circular consensus DNA sequencing libraries were constructed according to the manufacturers’ instructions. DNA sequencing was performed by the Scientific Operations core at the WSI on a Pacific Biosciences SEQUEL II (HiFi) instrument. Hi-C data were also generated from thorax tissue of ilPsePras1 using the Arima2 kit and sequenced on the Illumina NovaSeq 6000 instrument.

### Genome assembly, curation and evaluation

Assembly was carried out with Hifiasm ( Cheng *et al.*, 2021) and haplotypic duplication was identified and removed with purge_dups ( Guan *et al.*, 2020). The assembly was then scaffolded with Hi-C data ( Rao *et al.*, 2014) using YaHS ( Zhou *et al.*, 2023). The assembly was checked for contamination and corrected as described previously ( Howe *et al.*, 2021). Manual curation was performed using HiGlass ( Kerpedjiev *et al.*, 2018) and PretextView ( Harry, 2022). The mitochondrial genome was assembled using MitoHiFi ( Uliano-Silva *et al.*, 2023), which runs MitoFinder ( Allio *et al.*, 2020) or MITOS ( Bernt *et al.*, 2013) and uses these annotations to select the final mitochondrial contig and to ensure the general quality of the sequence.

A Hi-C map for the final assembly was produced using bwa-mem2 ( Vasimuddin *et al.*, 2019) in the Cooler file format ( Abdennur & Mirny, 2020). To assess the assembly metrics, the *k*-mer completeness and QV consensus quality values were calculated in Merqury ( Rhie *et al.*, 2020). This work was done using Nextflow ( Di Tommaso *et al.*, 2017) DSL2 pipelines “sanger-tol/readmapping” ( Surana *et al.*, 2023a) and “sanger-tol/genomenote” ( Surana *et al.*, 2023b). The genome was analysed within the BlobToolKit environment ( Challis *et al.*, 2020) and BUSCO scores ( Manni *et al.*, 2021; Simão *et al.*, 2015) were calculated.

Table 3 contains a list of relevant software tool versions and sources.

**Table 3.** Software tools: versions and sources.

Software tool	Version	Source
BlobToolKit	4.2.1	https://github.com/blobtoolkit/blobtoolkit
BUSCO	5.3.2	https://gitlab.com/ezlab/busco
Hifiasm	0.16.1-r375	https://github.com/chhylp123/hifiasm
HiGlass	1.11.6	https://github.com/higlass/higlass
Merqury	MerquryFK	https://github.com/thegenemyers/MERQURY.FK
MitoHiFi	3	https://github.com/marcelauliano/MitoHiFi
PretextView	0.2	https://github.com/wtsi-hpag/PretextView
purge_dups	1.2.5	https://github.com/dfguan/purge_dups
sanger-tol/genomenote	v1.0	https://github.com/sanger-tol/genomenote
sanger-tol/readmapping	1.1.0	https://github.com/sanger-tol/readmapping/tree/1.1.0
YaHS	1.2a.2	https://github.com/c-zhou/yahs

### Genome annotation

The BRAKER2 pipeline ( Brůna *et al.*, 2021) was used in the default protein mode to generate annotation for the *Pseudoips prasinana* assembly (GCA_951640165.1) in Ensembl Rapid Release at the EBI.

### Wellcome Sanger Institute – Legal and Governance

The materials that have contributed to this genome note have been supplied by a Darwin Tree of Life Partner. The submission of materials by a Darwin Tree of Life Partner is subject to the ‘Darwin Tree of Life Project Sampling Code of Practice’, which can be found in full on the Darwin Tree of Life website here. By agreeing with and signing up to the Sampling Code of Practice, the Darwin Tree of Life Partner agrees they will meet the legal and ethical requirements and standards set out within this document in respect of all samples acquired for, and supplied to, the Darwin Tree of Life Project.

Further, the Wellcome Sanger Institute employs a process whereby due diligence is carried out proportionate to the nature of the materials themselves, and the circumstances under which they have been/are to be collected and provided for use. The purpose of this is to address and mitigate any potential legal and/or ethical implications of receipt and use of the materials as part of the research project, and to ensure that in doing so we align with best practice wherever possible. The overarching areas of consideration are:

•       Ethical review of provenance and sourcing of the material

•       Legality of collection, transfer and use (national and international) 

Each transfer of samples is further undertaken according to a Research Collaboration Agreement or Material Transfer Agreement entered into by the Darwin Tree of Life Partner, Genome Research Limited (operating as the Wellcome Sanger Institute), and in some circumstances other Darwin Tree of Life collaborators.

## Data Availability

European Nucleotide Archive: *Pseudoips prasinana* (green silver-lines). Accession number PRJEB61694; https://identifiers.org/ena.embl/PRJEB61694 ( Wellcome Sanger Institute, 2023). The genome sequence is released openly for reuse. The *Pseudoips prasinana* genome sequencing initiative is part of the Darwin Tree of Life (DToL) project. All raw sequence data and the assembly have been deposited in INSDC databases. Raw data and assembly accession identifiers are reported in Table 1.

## Author information

Members of the University of Oxford and Wytham Woods Genome Acquisition Lab are listed here: https://doi.org/10.5281/zenodo.7125292.

Members of the Darwin Tree of Life Barcoding collective are listed here: https://doi.org/10.5281/zenodo.4893703.

Members of the Wellcome Sanger Institute Tree of Life Management, Samples and Laboratory team are listed here: https://doi.org/10.5281/zenodo.10066175.

Members of Wellcome Sanger Institute Scientific Operations: Sequencing Operations are listed here: https://doi.org/10.5281/zenodo.10043364.

Members of the Wellcome Sanger Institute Tree of Life Core Informatics team are listed here: https://doi.org/10.5281/zenodo.10066637.

Members of the Tree of Life Core Informatics collective are listed here: https://doi.org/10.5281/zenodo.5013541.

Members of the Darwin Tree of Life Consortium are listed here: https://doi.org/10.5281/zenodo.4783558.
