# Supplementary material for: The genome sequence of the Green Silver-lines, Pseudoips prasinana (Linnaeus, 1758)
Source: Wellcome Open Res. 2024 Mar 1;9:117. [Version 1] doi: 10.12688/wellcomeopenres.21014.1 (PMC11372352; doi:10.12688/wellcomeopenres.21014.1)
Supplement: Peer review history

# Reviewer report: Reviewer response for version 1

**DOI:** 10.21956/wellcomeopenres.23249.r94074

**Published:** 2024-09-03

**Authors:** Annabel Whibley

**Affiliations:** 1 The University of Auckland, Auckland, Auckland, New Zealand; 2 Bragato Research Institute, Grapevine Improvement, Lincoln, New Zealand

Douglas Boyes et.al report the sequencing, genome assembly and annotation of *Pseudoips prasinana *(a moth known the the Green Silver-lines, which reflect its forewing colour pattern). The natural history of this moth is engagingly presented and the genomic resources generated are of excellent quality, employed appropriate and well-documented methods with accessions available as stated in public databases.

 I second the suggestion of the previous reviewer to add "moth" to the title. 

 Additional minor comments relate to templated parts of the report that I have raised previously. Namely, (1) that the geographical co-ordinates should be noted as latitude and longitude in the Genome Sequence Report section; (2)  that kmer length should be reported for the MerquryFK evaluations and (3) that the bead:sample ratio used for AMPure cleanup should be 0.6x not 1.8x.

Are sufficient details of methods and materials provided to allow replication by others?

Yes

Is the rationale for creating the dataset(s) clearly described?

Yes

Are the datasets clearly presented in a useable and accessible format?

Yes

Are the protocols appropriate and is the work technically sound?

Yes

Reviewer Expertise:

Genomics, Evolution, Bioinformatics

I confirm that I have read this submission and believe that I have an appropriate level of expertise to confirm that it is of an acceptable scientific standard.

# Reviewer report: Reviewer response for version 1

**DOI:** 10.21956/wellcomeopenres.23249.r92629

**Published:** 2024-08-22

**Authors:** Jeffrey Marcus

**Affiliations:** 1 University of Manitoba, Winnipeg, Manitoba, Canada

In this manuscript, the authors describe the sequencing and assembly of the *Pseudoips prasinana *genome using DNA from an adult female specimen collected in the UK. The primary genome sequence assembly includes proposed chromosomal pseudomolecule sequences for 31 autosomes, the Z sex chromosome, the W sex chromosome and a complete mitochondrial genome. On the whole, this is a useful contribution to the scientific literature, but please see my comments below regarding the identification of the specimen, and details of mitogenome assembly.

**Some suggestions to the authors:**

1. **Title:** I recommend that the title be amended to “The genome sequence of the Green Silver-lines Moth *Pseudoips prasinana* (Linnaeus, 1758)”. Unless they are intimately familiar with the common and scientific names of the moths of Britain, the common name “Green Silver-lines” will be obscure to most readers of this work.
2. **Method of Specimen identification:** The individual researcher who did the specimen identification was named, but keys/species descriptions consulted, or the morphological characters used for the identification have not been included in the manuscript.
3. The authors describe how “The mitochondrial genome was assembled using MitoHiFi (Uliano-Silva et al. 2022) which runs MitoFinder (Allio et al. 2020) or MITOS (Bernt et al. 2013) and uses these annotations to select the final mitochondrial contig…”. The authors do not describe which of the algorithms generated the final contig for the mitogenome presented in their work.

Are sufficient details of methods and materials provided to allow replication by others?

Partly

Is the rationale for creating the dataset(s) clearly described?

Yes

Are the datasets clearly presented in a useable and accessible format?

Yes

Are the protocols appropriate and is the work technically sound?

Yes

Reviewer Expertise:

Evolutionary biology of insects, phylogenomics

I confirm that I have read this submission and believe that I have an appropriate level of expertise to confirm that it is of an acceptable scientific standard.
